# Phosphohistone H3 (pHH3) is a prognostic and epithelial to mesenchymal transition marker in diffuse gliomas

**DOI:** 10.18632/oncotarget.7154

**Published:** 2016-02-03

**Authors:** Ping Zhu, Chuan-Bao Zhang, Pei Yang, Jing Chen, Yu-Qing Liu, Hui-Min Hu, Hua Huang, Zhao-Shi Bao, Wei Zhang, Wei-Jia Kong, Tao Jiang

**Affiliations:** ^1^ Department of Otolaryngology, Union Hospital, Tongji Medical College, Huazhong University of Science and Technology, Wuhan 430022, China; ^2^ Department of Molecular Neuropathology, Beijing Neurosurgical Institute, Capital Medical University, Beijing 100050, China; ^3^ Department of Neurosurgery, Beijing Tiantan Hospital, Capital Medical University, Beijing 100050, China; ^4^ Center of Brain Tumor, Beijing Institute for Brain Disorders, Beijing 100069, China; ^5^ China National Clinical Research Center for Neurological Diseases, Beijing 100050, China

**Keywords:** glioma, pHH3, prognosis, EMT

## Abstract

The World Health Organization (WHO) grading of gliomas stratifies tumors by histology. However, the aggressiveness of tumors in each grade still shows great heterogeneity. Phosphohistone H3 (pHH3) has been reported as an accurate marker of cells within the mitotic phase of the cell cycle in many kinds of cancers. To evaluate the role of pHH3 in predicting patient outcome and to annotate the functions of pHH3 in WHO grade II-IV gliomas, we analyzed the expression pattern of pHH3 and pHH3 associated genes by IHC and mRNA expression profiling. Phosphohistone H3, mRNA enrichment of histone H3 and associated gene signature all showed prognostic value in adult diffuse gliomas. Gene set enrichment analysis suggested that the expression of pHH3 had positive correlation with both epithelial to mesenchymal transition and immune response. These findings suggest that subgroups of diffuse gliomas defined by pHH3 and pHH3 signatures possess distinctive prognostic and biological characteristics.

## INTRODUCTION

Diffuse gliomas represent the vast majority of adult intracranial tumors and are divided into three histopathologic grades according to the latest World Health Organization (WHO) brain tumor classification system [1]. WHO grade III, also known as anaplastic diffuse gliomas, are characterized by histological (astrocytic, oligodendrocytic) and molecular varieties (IDH1/2, ATRX, TERT promoter mutation, 1p/19q deletion, etc.) [2]. The WHO classification, based solely on morphological features [3], may be supplemented with defined molecular aberrations (molecular classifications, gene mutations) [4–8]. This might help resolve the discrepancy between classification and clinical outcome [9, 10].

Phosphohistone H3 (pHH3), a sensitive marker for mitotic figure detection, has been widely reported in a variety of cancers [11–23]. An advantage of the immunohistochemical staining of pHH3 in mitoses counting is that the assessment is based on both the identification of positively stained figures as well as the morphologic confirmation of chromatin condensation [23]. This minimizes interobserver and inter-laboratory technical variations [12], and allows the determination of a more objective prediction of patient outcome. The impact of the conventional WHO grade and mitotic index on WHO grade II-IV diffuse gliomas followed by functional annotation based on high throughput datasets is not well established, and further efforts to integrate molecular markers may improve stratification of patient risk groups.

The main purpose of this study was to evaluate the role of pHH3 in predicting patient outcome and to annotate pHH3 functions in WHO II-IV diffuse gliomas. To achieve this, we first assessed the expression pattern of pHH3 in anaplastic diffuse glioma by immunohistochemistry. And by analyzing the matched mRNA expression profile data, we then obtained prognostic signatures and pHH3 associated subtypes to further interpret pHH3 functions in diffuse gliomas.

## RESULTS

### Phosphohistone H3 as an independent prognostic marker in anaplastic diffuse gliomas

We included sixty-one anaplastic diffuse gliomas from Chinese Glioma Tissue Database (CGTD) for immunohistochemical (IHC) staining. There were fourteen anaplastic astrocytomas (AA), seventeen anaplastic oligodendrogliomas (AO) and thirty anaplastic oligoastrocytomas (AOA). Patient age ranged from 18 to 74 years overall. Adjuvant treatment data were available for fifty-seven patients, of which fifty received radiation and thirty-five received chemotherapy. Nineteen patients received either radiation or chemotherapy, thirty-three patients received both, and five did not receive adjuvant treatment.

**Figure 1:**
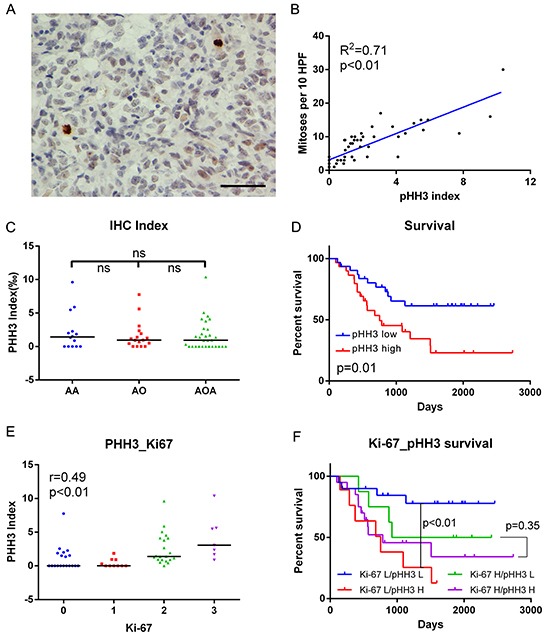
IHC data in anaplastic diffuse gliomas **A.** Examples of pHH3 positively stained mitotic figures (brown) shown at high magnification (Two positive staining mitotic figures in 576 cells in this field; bar, 50μm). **B.** pHH3 index significantly correlated with mitoses per 10 high power fields (HPF, linear regression, R^2^=0.71, p<0.01, N=61). **C.** PHH3 index showed no significant difference among histologies. Black bar, median index. **D.** Patients with high pHH3 (no lower than median, N=31) had poorer survival than those with low pHH3 (N=30). **E.** The expression of pHH3 was positively correlated with that of Ki-67 (Spearman's correlation, r=0.49, p<0.01). **F.** Effects of pHH3 and Ki-67 on survival of diffuse glioma patients (Ki-67 L/pHH3 L N=20, Ki-67 L/pHH3 H N=9, Ki-67 H/pHH3 L N=8, Ki-67 H/pHH3 H N=20). IHC, immunohistochemistry; AA, anaplastic astrocytoma; AO, anaplastic oligodendroglioma; AOA, anaplastic oligoastrocytoma; ns, not significant; L, low mitotic index/low expression; H, high mitotic index/high expression.

To characterize the proliferative features of those samples, we quantified the mitotic index using pHH3 immunostaining in all the 61 samples. As shown in the representative examples, pHH3 staining positive mitotic figures can be easily identified (Figure 1A). The index was calculated as mitotic figures divided by total tumor cells (>1000) in several 400× high cellular fields. The index ranged from 0 to 10.37‰, with a median of 0.97‰, which was used as the cut-off the high (≥0.97‰) and low (<0.97‰) proliferative tumors in the following analysis. The index significantly correlated with mitoses per 10 high power fields (HPF, Figure 1B, linear regression, R^2^=0.71, p<0.01). There was no statistical difference among the three types of histology, although AA samples showed slightly higher median and average indices (Figure 1C). The high proliferative tumors showed worse prognosis (p = 0.01, Figure 1D).

To further characterize relationships between classical proliferative markers and mitotic index, IHC for Ki-67 was performed in cases with enough tissue samples. In these samples (57/61), these two markers showed significant correlation (r=0.49, p<0.01, Figure 1E). And with the use of the mitotic index, there was no prognostic difference between high- versus low-index tumors in the Ki-67 high subset (p = 0.35, Figure 1F). In contrast, the effect of mitotic index on Ki-67 low tumors was more substantial (p<0.01, Figure 1F).

To further clarify the relationships between variables and patient outcome, univariate and multivariate Cox analysis was performed. When the entire cohort was examined by univariate Cox analysis, mitotic index, age at initial diagnosis, IDH mutation status, radiotherapy and chemotherapy were all statistically significant or nearly significant (Table 1). This held true except for age after multivariate Cox analysis, which showed the independent prognostic value of mitotic index (p = 0.04, Table 1).

**Table 1:** Univariate and multivariate Cox analysis of pHH3 in anaplastic gliomas

Variables	Univariate				Multivariate			
	HR	95% CI		p	HR	95% CI		p
		Lower	Upper			Lower	Upper	
pHH3 index†	1.26	1.10	1.45	<0.01	1.19	1.01	1.41	0.04
Age†	1.03	1.00*	1.06	0.03	1.02	0.99	1.05	0.31
IDH1/2 status(Mut/WT)	0.33	0.15	0.73	0.01	0.11	0.03	0.36	<0.01
Radiotherapy(Y/N)	0.38	0.14	1.03	0.06	0.13	0.03	0.57	0.01
Chemotherapy(Y/N)	0.39	0.19	0.83	0.01	0.15	0.05	0.45	<0.01

HR, hazard ratio; CI, confidence interval; Mut, mutation; WT, wild type; Y, received therapy; N, not received therapy;

† pHH3 index and age were analyzed as continuous variables.

* 95% CI here was 1.0022-1.0581. Cases with this information available: pHH3 index, N=61; Age, N=61; IDH1/2, N=52; Radiotherapy, N=57; Chemotherapy: N=57. Endpoint: overall survival.

### Enrichment of genes encoding histone H3 as a prognostic marker in glioblastoma multiforme (GBM)

**Figure 2:**
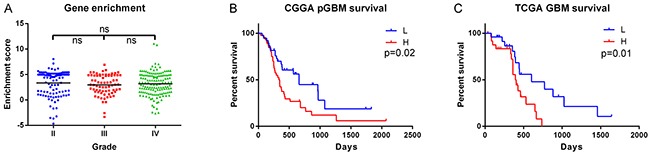
Enrichment of genes encoding histone H3 in RNAseq data **A.** Histone H3 coding genes showed no significant difference among grades in CGGA data (Grade II, N=109; Grade III, N=72; Grade IV, N=144). **B.** The enrichment score showed prognostic value in CGGA primary GBM (pGBM) patients (L group, N=38; H group N=46). **C.** The enrichment score showed prognostic value in the young GBM patients (age<55) from TCGA dataset (L group, N=25; H group, N=25). ns, not significant; L, low enrichment score group; H, high enrichment score group.

RNA sequencing data of 325 WHO grade II-IV diffuse glioma samples from CGGA (109 grade II samples, 72 grade III samples and 144 grade IV samples) and 169 GBM samples from TCGA were downloaded to evaluate the prognostic effect of the genes encoding histone H3. We analyzed the enrichment score of genes encoding histone H3 (Supplementary Table S1) [24] by GSEA algorithm. GSEA is a computational method that assesses coordinate expression changes at a gene set level [25]. There was no significant difference between every two grades of diffuse gliomas (Figure 2A). In the 84 primary GBM samples (median age: 52 years) with follow-up in CGGA data, tumors with high enrichment scores (higher than median) were clinically more aggressive (Figure 2B). For the grade II and grade III glioma samples in CGGA dataset, the enrichment score showed no significant prognostic value (data not shown). In contrast, in the 158 GBM with follow-up from TCGA dataset, this held true when only young patients were included (50 patients, age <55, median age: 48.6 years, Figure 2C).

### Phosphohistone H3 associated gene signature as an independent prognostic marker in GBM

**Figure 3:**
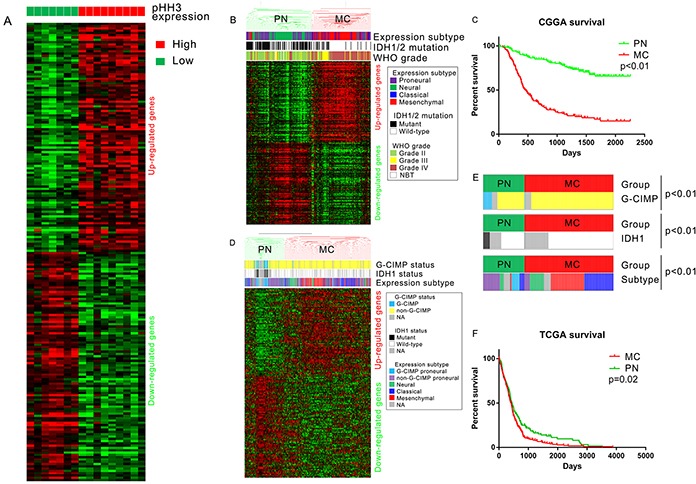
PHH3 signature derived from mRNA expression microarray data **A.** Differential analysis of pHH3 high group (N=9) versus pHH3 low group (N=7). The top 100 differently expressed probes were included in this figure. **B.** Hierarchical clustering of CGGA mRNA microarray data (305 glioma samples and 5 normal brain tissues) by pHH3 signature (top 200 differently expressed genes, 100 up-regulated and 100 down-regulated). **C.** Prognostic difference of subtypes defined by pHH3 signature: the overall survival of PN subtype(median survival not reached, median follow-up 1330 days) was markedly better than that of MC subtype(median survival 462 days, p<0.01; PN: N=162, MC: N=148). **D.** Hierarchical clustering of TCGA GBM mRNA microarray data (N=603) by pHH3 signature (same genes with those in Figure 3B). **E.** Distribution of G-CIMP, IDH1, Group subtype within each pHH3 expression subgroup (chi-square test). **F.** Prognostic difference of subtypes defined by pHH3 signature in TCGA GBM dataset (PN: N=191, MC: N=412). PN, proneural-neural subtype; MC, mesenchymal-classical subtype; NBT, normal brain tissue.

Sixteen of the sixty-one samples (2 AA, 7AO, 7AOA) with pHH3 staining were also analyzed by mRNA expression microarray (seven, low mitotic index; nine, high mitotic index). To gain insights into the significance of pHH3 in diffuse gliomas, we performed Significance Analysis of Microarrays (SAM) for differential analysis. There were 4796 up-regulated probes (3397 genes) and 2011 down-regulated probes (1590 genes) in high mitotic index group with FDR<0.05 after 100 times of permutation test (Figure 3A). This analysis revealed the distinct expression patterns in each of the groups.

To determine if these genes have prognostic value, we built a classification scheme (pHH3 signature) of the top 200 differently expressed probes [172 genes, 100 up-regulated (83 genes) and 100 down-regulated (89 genes), Supplementary Table S2]. Messenger RNA expression microarray data and clinical information of 5 normal brains and 305 WHO grade II-IV diffuse gliomas were downloaded from CGGA database. TCGA expression subtype [26] of these samples were annotated by Prediction Analysis of Microarrays (PAM) [5, 27]. We utilized hierarchical clustering to assign tumors to subclasses according to the expression pattern of pHH3 signature genes (200 probes, 172 genes, Figure 3B). The names of the groups were defined to recognize the dominant feature of the TCGA expression subtypes. The mesenchymal-classical (MC) subtype had a vast majority of WHO grade III-IV (133/148), wild typed IDH1 (122/148), mesenchymal and classical tumors (125/148), while all the five normal brain tissues, most WHO grade II tumors (111/126) were in proneural-neural (PN) subtype. Kaplan-Meier plots for cases in the CGGA dataset showed that median survival of the PN subtype(median survival not reached, median follow-up 1330 days) was markedly longer than MC subtype(median survival 462 days, median follow-up 443 days, Figure 3C, p<0.01).

To assess the prognostic value of pHH3 signature in WHO grade II-III gliomas, hierarchical clustering of 177 WHO II-III glioma samples out of the 310 microarray data was used to assign samples into pHH3 subtypes (PN or MC). The majority of WHO grade II (110/126, chi-square test, p<0.01) and IDH1/2 mutated (93/112, chi-square test, p<0.01) samples were clustered into PN subtype (Supplementary Figure S1A). Patients in PN subtype showed better overall survival than those in MC subtype (Supplementary Figure S1B, p<0.01).

We further downloaded mRNA expression microarray data and clinical information of 603 GBM from TCGA database to determine if this classification scheme has prognostic value independent of tumor grade. Hierarchical clustering was repeated in these WHO grade IV samples according to the expression pattern of pHH3 signature genes (152 genes overlapped with pHH3 signature genes, Figure 3D). The analysis also revealed that this large number of GBM samples could be classified into groups that differed markedly in their expression of these genes. A significant association was also seen between group and molecular subtype: G-CIMP negative tumors, IDH1 wild type tumors, mesenchymal and classical tumors were clustered into MC subtype, while the other tumors were enriched in PN subtype (Figure 3E). The prognosis of MC subtype (median overall survival 415 days, median follow-up 326 days) was inferior to that of PN subtype (median overall survival 434 days, median follow-up 322 days, p = 0.02, Figure 3F).

To deeply assess the prognostic value of pHH3 associated gene signature in GBM, we performed Cox analysis in the TCGA GBM microarray dataset (Supplementary Table S3). On univariate analysis, the gene signature was significantly associated with survival along with IDH1 status, patient age, MGMT promoter methylation, radiotherapy and chemotherapy. On multivariate analysis, only age, IDH1 status, radiation and chemotherapy remained to be significant. Cox analysis was also performed in CGGA microarray dataset (Table 2), pHH3 subtype was significantly associated with survival (p<0.01) along with IDH1/2 mutation, patient age, WHO grade, radiotherapy and chemotherapy (nearly significant, p = 0.08). On multivariate analysis, the association was also significant (p<0.01) after adjusting for all the other factors included. Thus, the classification scheme showed prognostic value independent of tumor grade, IDH1/2 status, patient age and therapies (not independent of IDH1 status, age, MGMT promoter methylation and therapies in TCGA GBM samples).

**Table 2:** Univariate and multivariate Cox analysis of pHH3 in CGGA microarray data

Variables	Univariate				Multivariate			
	HR	95% CI		p	HR	95% CI		p
		Lower	Upper			Lower	Upper	
pHH3 signature group (H/L)	5.08	3.57	7.23	<0.01	2.35	1.44	3.83	<0.01
IDH1/2 status (Mut/WT)	0.33	0.23	0.46	<0.01	1.08	0.69	1.69	0.74
Age (Continuous variable)	1.04	1.03	1.06	<0.01	1.01	0.998	1.03	0.08
Grade (IV/III/II)	3.14	2.54	3.88	<0.01	2.52	1.92	3.32	<0.01
Radiation (Y/N)	0.60	0.41	0.89	0.01	0.39	0.25	0.61	<0.01
Chemotherapy (Y/N)	1.33	0.96	1.82	0.08	0.71	0.50	1.02	0.06

Samples with overall survival available (N=303) were included. HR, hazard ratio; CI, confidence interval; H, high expression; L, low expression; Mut, mutation; WT, wild type; Y, received therapy; N, not received therapy.

### Functional annotation of phosphohistone H3

**Figure 4:**
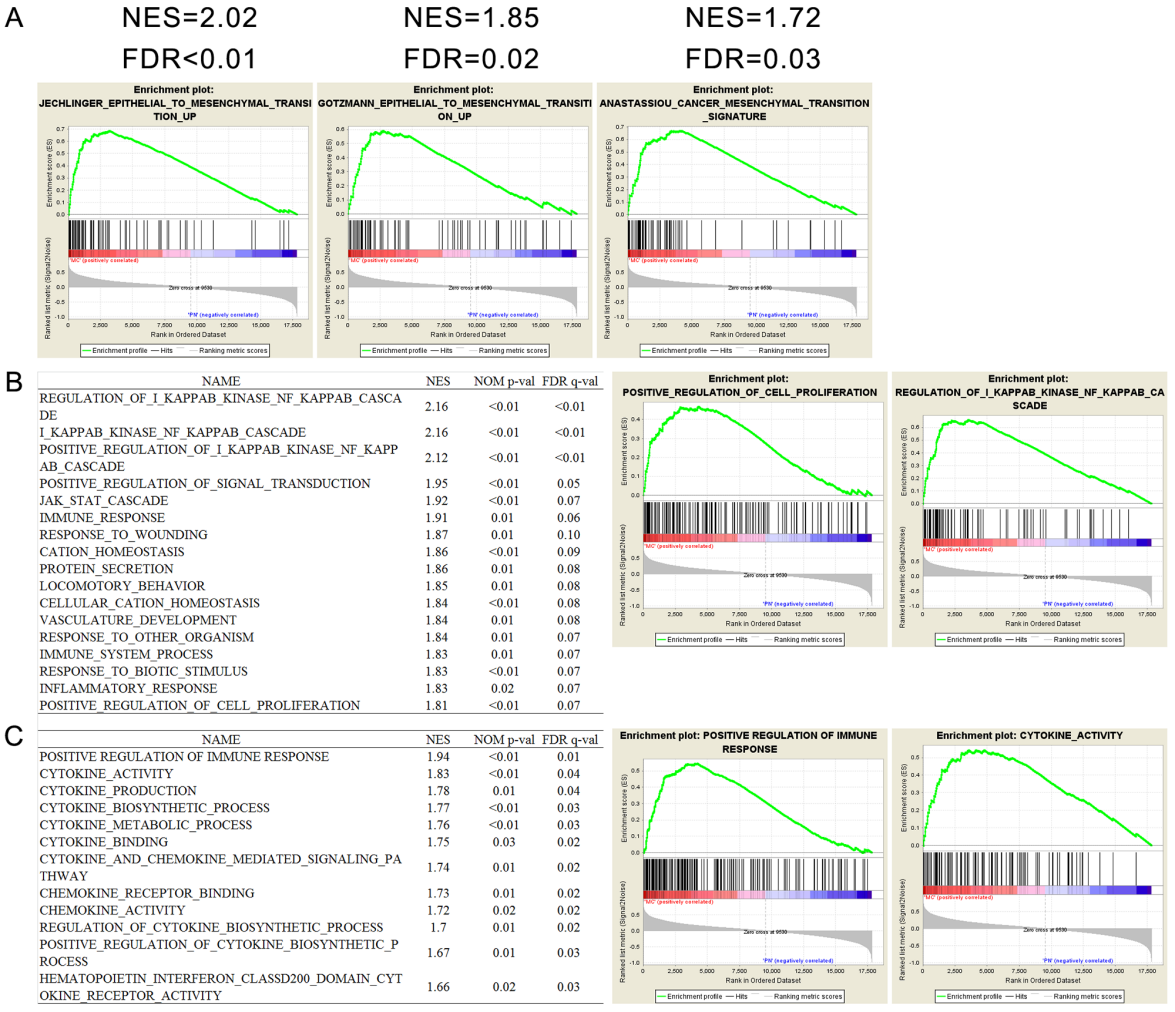
Functional annotation of pHH3 in TCGA GBM microarray data (N=603) **A.** Gene Set Enrichment Analysis (GSEA) showed enrichment of EMT (epithelial to mesenchymal transition) related genes in patients classified by pHH3 signature. **B.** Biological processes enriched in pHH3 subtypes and representative enrichment plots. **C.** GSEA analysis of immune associated gene sets and representative enrichment plots. NES, normalized enrichment score; FDR, false discovery rate; NOM *p* value, nominal *p* value; MC, mesenchymal-classical subtype; PN, proneural-neural subtype; All the gene sets were downloaded from Molecular Signature Database (MSigDB) in GSEA website.

The results above indicated the subtype preference and epithelial to mesenchymal transition(EMT) association of pHH3. To confirm whether MC samples were enriched with EMT related pathways, we performed GSEA in TCGA GBM cases (N=603), as well as in CGGA microarray data (177 WHO grade II-III samples). EMT related gene sets were obtained using the GSEA tool from MIT (www.broad.mit.edu/gsea, Supplementary Table 4). Several gene sets of EMT were associated with pHH3 subtypes (Figure 4A, Supplementary Figure S1C). Collectively, these data suggest that MC phenotype was enriched by genes of EMT, whereas PN phenotype correlated strongly with the decrease of genes of EMT.

Phosphohistone H3 has been widely reported as an accurate marker to identify cells within the proliferating phase of the cell cycle [11–23]. To gain insights into the biological consequences of pHH3 in diffuse gliomas, we next performed GSEA with biological processes associated gene sets. As expected, in the 603 TCGA GBM cases, the MC subtype was significantly associated with proliferation. Moreover, a large number of immune associated biological processes showed significant correlation (Figure 4B). To validate the immune association of pHH3, we then downloaded eleven immune related gene sets from MSigDB, which all showed significant correlation with pHH3 subtypes (Figure 4C).

These findings indicated that the expression of pHH3 also had positive correlation with both EMT and immune response besides proliferation.

## DISCUSSION

The severity of diffuse gliomas, common primary tumors in the adult CNS, is classified by malignancy grades (II to IV) according to features of cellular atypia, cell proliferation, angiogenesis, and necrosis [3]. The relative subjectivity and inconsistency in its designation cause bias in diagnosis, which will result in clinical confusion, generate artificial heterogeneity and complexities in diffuse glioma investigations, and hinder the development of targeted therapies [10]. To date, a growing number of definitive histopathologic, biologic, or molecular markers have been identified to separate the patients who may need more aggressive therapy or follow-up from those who does not [4, 8, 28, 29].

Ki-67 and pHH3 have long been accepted as cellular proliferation markers in a great variety of cancers [11-23, 30, 31]. PHH3 staining is believed to be a useful diagnostic complementary tool to standard H&E mitotic count. It can be easily identified and can minimize interobserver and inter-laboratory technical variations [31]. In the present study, by IHC staining, we investigated the prognostic value of pHH3 in anaplastic diffuse gliomas stratified by Ki-67 expression. We found that the effect of pHH3 on patient outcome was small in the high Ki-67 expression subset of tumors, while it was more substantial in low Ki-67 expression subset (Figure 1F). The results above suggested that the prognostic impact of mitotic index might be conditional on Ki-67 expression level. More studies are needed to clarify this issue. IDH1/2 mutation has been reported to have significant association with mitotic index, and the prognostic value of mitotic index varied according to IDH1/2 status [11]. We proved that pHH3 signature was significantly associated with IDH1 mutation status (Figure 3E) and the prognostic value of pHH3 was independent of WHO grade, IDH1/2 status, patient age and therapies.

Although dozens of studies have given evidence of pHH3 expression contributing to mitosis, there are few ones trying to interpret pHH3 function precisely. Here in this study, by integrating IHC data and high through-put data (total sample size: 1468. 61 IHC samples, 325 RNA sequencing samples from CGGA, 169 RNA sequencing samples from TCGA, 310 mRNA microarray samples from CGGA and 603 mRNA microarray samples from TCGA), we demonstrated the significant correlation between pHH3 and genes in EMT pathways, as well as those associated with immune response. These functional annotations, in addition to proliferation association, should also be validated in other independent datasets. EMT is considered to play an essential role in the progression and metastasis of solid tumors [32]. The positive correlation of pHH3 and EMT genes well explained the aggressiveness of pHH3 high diffuse gliomas.

In summary, we have used the mitosis marker pHH3 to identify mitoses in anaplastic diffuse gliomas. The mitotic index was a prognostic marker especially in low Ki-67 expression cases. In multivariate analysis, the pHH3-mitotic index was an independent predictor of survival after adjusting for relevant clinical variables. By integrating IHC data and high through-put data, pHH3 showed significant correlation with epithelial to mesenchymal transition pathways. These findings suggest that stratification of diffuse gliomas into subsets defined by the pHH3 or pHH3 signatures leads to subgroups with distinct prognostic and biological characteristics.

## MATERIALS AND METHODS

### Patients and samples

Sixty-one anaplastic diffuse glioma samples from the CGTD, were included in this study (Some of them were included in a retrospective study [33], but pHH3 was not tested then). Tumor tissue samples were obtained by surgical resection during January 2006 through December 2012. The patients were then treated by radiotherapy and/or chemotherapy with alkylating agents. The histological diagnoses were confirmed by two neuropathologists according to the 2007 WHO classification guidelines. Only samples with >80% tumor cells were enrolled for analysis. The study was approved by the Institutional Review Boards of all hospitals involved, and written informed consents were obtained from all patients.

RNA sequencing data of 325 WHO grade II-IV diffuse gliomas from Chinese Glioma Genome Atlas(CGGA, http://www.cgga.org.cn) and 169 GBM from the Cancer Genome Atlas (TCGA, http://cancergenome.nih.gov) were involved for enrichment analysis and validation of genes encoding histone H3. The clinical and molecular information (IDH1/2 mutation, MGMT promoter methylation, etc.) of TCGA samples were also downloaded [8]. Whole genome mRNA expression microarray data of 305 WHO grade II-IV diffuse gliomas and five normal brain tissues from CGGA and 603 GBMs from TCGA were enrolled for functional annotation and validation of the results.

### Proliferation assessment by standard H&E, pHH3 and Ki-67 staining

Mitotic count per 10 HPF was determined by standard H&E staining under 10 high magnification (×400) microscopic fields as previously reported [14].

Immunoperoxidase staining for phosphohistone H3 (pHH3)(Serine 10 and Serine 28) was performed following the standard protocol recommended by the manufacturer (RAB-0693, working solution, Fuzhou Maixin Biotech. Co., Ltd.). The index was calculated as mitotic figures divided by total tumor cells (>1000) in several 400× high cellular fields [11]. Two experienced neuropathologists manually counted mitoses per over 1000 tumor nuclei in the highest mitotically active foci. Only nuclei accompanied by chromatin condensation were counted, while the uniformly stained positive nuclei and the finely speckled stained nuclei were excluded [22].

Immunohistochemistry was performed in 57 of the 61 anaplastic glioma samples (four cases without enough tissue samples) to detect Ki-67 protein expression following the manufacturer's protocol (sc-15402, dilution: 1:200, Santa Cruz Biotechnology, Santa Cruz, CA). Each slide was individually reviewed and scored by two experienced neuropathologists, using a four-grade scale from 0 to 3, with 0=no or <5% occurrence of nuclear staining, 1 = 5-10% of nuclei of tumor cells positively stained, 2 = 10–30 % of nuclei of tumor cells positively stained, 3 = >30 % of nuclei of tumor cells positively stained. Score 2 and 3 (>10% of nuclei of tumor cells positively stained) were defined as high Ki-67 expression.

Controls without primary antibody and positive control tissues were included in all experiments to ensure the quality of the staining.

### RNA extraction and mRNA expression microarray

Sixteen of the 61 anaplastic glioma samples were also analyzed by mRNA expression microarray. Total RNA of snap frozen samples were extracted using RNeasy Mini Kit (Qiagen) according to the manufacturer's instructions. RNA intensity was checked using 2100 Bioanalyzer (Agilent Technologies) and only high quality samples with RNA Integrity Number (RIN) value greater than or equal to 7.0 were used for mRNA expression microarray. The microarray analysis was performed as previously reported [5] using the Agilent Whole Human Genome Array according to the manufacturer's instructions.

### DNA pyro-sequencing for IDH1/2 mutation

DNA pyro-sequencing for IDH1/2 mutation in 52 of the 61 anaplastic glioma samples was performed as previously reported [34–36]. In brief, the genomic regions spanning R132 of IDH1 and R172 of IDH2 were PCR amplified and subjected to pyrosequencing on PyroMark Q96 ID System (QIAGEN) using the primers as reported.

### Statistical analysis

Overall survival was calculated from the date of histological diagnosis to the date of either death or last follow-up. The survival curve was calculated using the Kaplan–Meier method and the difference was analyzed using the two sided log-rank test. Cox regression analysis was used to evaluate the prognostic value of the markers. Differential expression analysis of microarray data was performed using SAM (http://www-stat.stanford.edu/~tibs/SAM). Student's t-test was used for comparison between two groups. A p value <0.05 was considered statistically significant. All the analyses were performed in R (version 3.1.1) and GraphPad Prism (version 6.0.1) for Windows.

## SUPPLEMENTARY FIGURE AND TABLES








