# Supplementary material for: Phosphohistone H3 (pHH3) is a prognostic and epithelial to mesenchymal transition marker in diffuse gliomas
Source: Oncotarget. 2016 Feb 3;7(29):45005–14. doi: 10.18632/oncotarget.7154 (PMC5216701; doi:10.18632/oncotarget.7154)
Supplement: Supplementary file 1 [file oncotarget-07-45005-s001.pdf]

SUPPLEMENTARY FIGURE AND TABLES

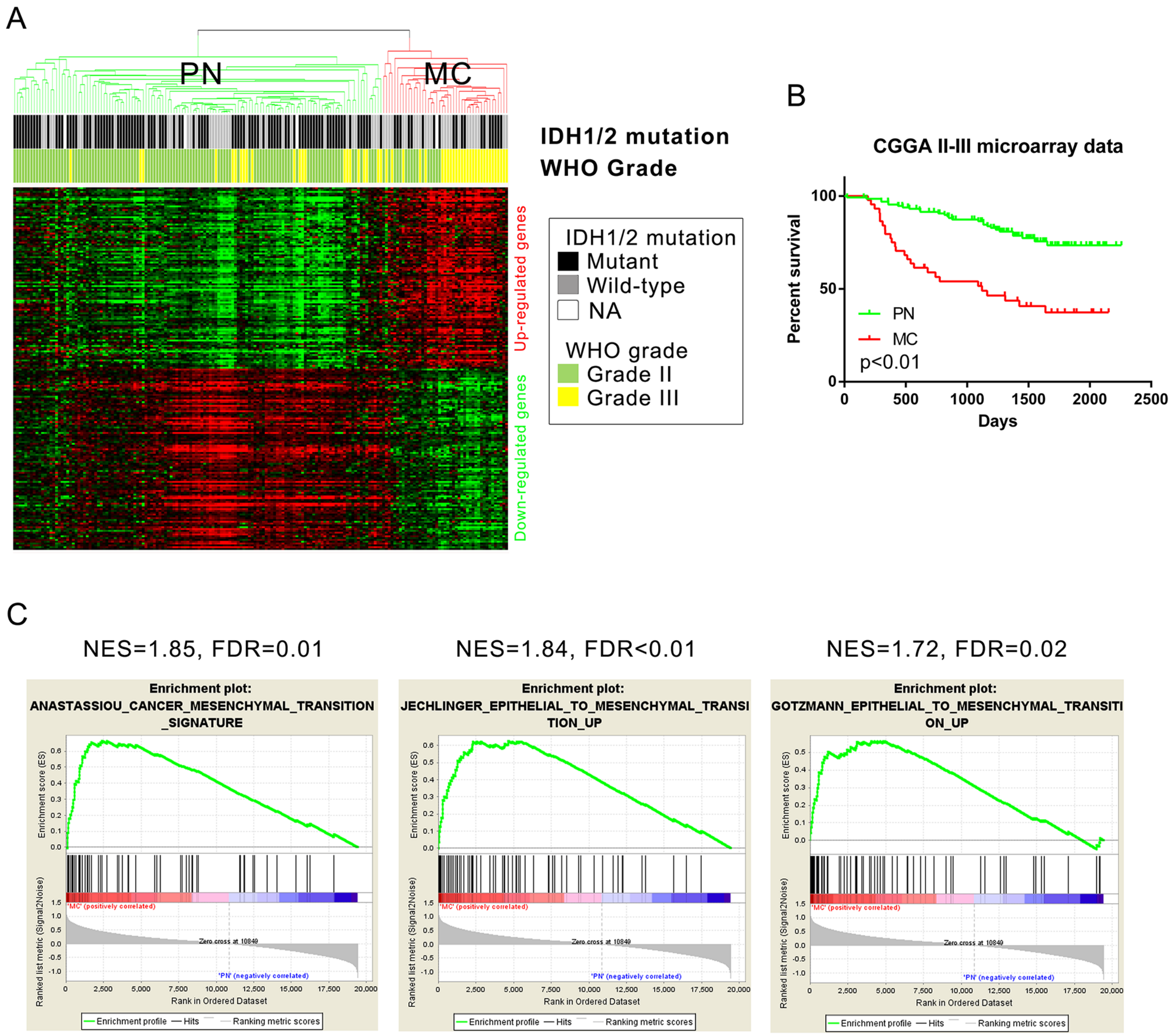

Supplementary Figure 1: PHH3 signature and functional annotation in WHO grade II-III gliomas. A. Hierarchical clustering of 177 CGGA WHO grade II-III glioma mRNA microarray data by pHH3 signature. More grade II (110/126, chi-square test, p<0.01) and IDH1/2 mutated samples (93/112, chi-square test, p<0.01) were clustered in PN subtype. **B.** PN subtype (median survival not reached, median follow-up 1412 days) had a longer overall survival than MC subtype (median survival 1121 days, log-rank test, p<0.01). **C.** GSEA showed enrichment of EMT related genes in WHO grade II-III patients classified by pHH3 signature. PN, proneural-neural subtype; MC, mesenchymal-classical subtype; NES, normalized enrichment score; FDR, false discovery rate.

Supplementary Table S1: Eighteen genes encoding histone H3.

See Supplementary File 1

Supplementary Table S2: Top 200 differently expressed probes between pHH3 low samples and pHH3 high samples.

See Supplementary File 2

Supplementary Table S3: Univariate and multivariate Cox analysis of pHH3 in TCGA GBM microarray dataset

Variables	Univariate				Multivariate			
	HR	95% CI		p	HR	95% CI		p
		Lower	Upper			Lower	Upper	
pHH3 signature group (H/L)	1.29	1.04	1.60	0.02	0.86	0.62	1.20	0.38
IDH1 status (Mut/WT)	0.38	0.23	0.62	<0.01	0.46	0.24	0.86	0.02
Age (Continuous variable)	1.04	1.03	1.04	<0.01	1.03	1.01	1.04	<0.01
MGMT promoter methylation (M/U)	0.72	0.56	0.92	<0.01	0.91	0.68	1.20	0.50
Radiation (Y/N)	0.24	0.12	0.47	<0.01	0.29	0.12	0.67	<0.01
Chemotherapy (Y/N)	0.37	0.29	0.45	<0.01	0.39	0.27	0.54	<0.01

HR, hazard ratio; CI, confidence interval; H, high expression; L, low expression; Mut, mutation; WT, wild type; M, methylated; U, unmethylated; Y, received therapy; N, not received therapy. Five hundred and thirty-three samples with overall survival information were included in this analysis.

Supplementary Table S4: Epithelial to mesenchymal transition gene signatures.

See Supplementary File 3
